# New Constitutive Matrix in the 3D Cell Method to Obtain a Lorentz Electric Field in a Magnetic Brake

**DOI:** 10.3390/s18103185

**Published:** 2018-09-20

**Authors:** José Miguel Monzón-Verona, Pablo Ignacio González-Domínguez, Santiago García-Alonso

**Affiliations:** 1 Electrical Engineering Department, University of Las Palmas de Gran Canaria, 35017 Las Palmas de Gran Canaria, Spain; josemiguel.monzon@ulpgc.es; 2 Institute for Applied Microelectronics, University of Las Palmas de Gran Canaria, 35017 Las Palmas de Gran Canaria, Spain; 3 Department of Electronic Engineering and Automatics (DIEA), University of Las Palmas de Gran Canaria, 35017 Las Palmas de Gran Canaria, Spain; santiago.garciaalonso@ulpgc.es

**Keywords:** cell method, Hall sensors, Lorentz electric field, magnetic brake

## Abstract

In this work, we have obtained a new constitutive matrix to calculate the induced Lorentz electric current of in a conductive disk in movement within a magnetic field using the cell method in 3D. This disk and a permanent magnet act as a magnetic brake. The results obtained are compared with those obtained with the finite element method (FEM) using the computer applications Getdp and femm. The error observed is less than 0.1173%. Likewise, a second verification has been made in the laboratory using Hall sensors to measure the magnetic field in the proximity of the magnetic brake.

## 1. Introduction

The use of magnetic brakes has obvious advantages compared to brakes based on mechanical friction. The mechanical friction brakes have the risks of hydraulic fluid loss or the contamination of the fluid by cooling water, with a consequent loss of braking power, among others [1]. The use of permanent magnets in linear magnetic brakes is explained in detail in [2]. In [1], a mixed system with mechanical friction and a magnetic brake is analyzed, comparing the results obtained with an analytical equation and the finite element method (FEM) in 2D and 3D.

In most of the works consulted in the bibliography, approximate analytical equations or numerical methods such as the FEM are used. In the present work, we propose the finite formulation (FF) [3], and the cell method (CM) as its associated numerical method [4,5], to analyze this type of device.

FF works with global magnitudes associated with spatially oriented elements such as volumes, surfaces, lines and points of the discretized space, as well as temporal elements, instead of field magnitudes associated with the independent variables of spatial and temporal coordinates [6]. In FF, the equations of constitutive type—equations of the medium—are clearly differentiated from the topological type—equations of balance. The analysis of the magnetic brake with this methodology facilitates, to a great extent, the boundary conditions and continuity, when working with global magnitudes.

The contribution of this work consists on obtaining a new constitutive equation that relates the Lorentz current with the magnetic potential using CM. This result applies to the magnetic brake. This consists of a copper disk with an angular velocity $\vec{W_{r}}$, and a permanent magnet is placed in front it.

The structure of this paper is as follows. In Section 2, the methodology to obtain the constitutive matrix in the CM and the corresponding Lorentz term is explained in detail. Section 3 explains the results obtained and their comparison with other references. Finally, in Section 4, the conclusions are given.

## 2. The Constitutive Matrix in the Cell Method

The electric field strength, $\vec{E}$, measured in a fixed coordinate system with respect to the laboratory, is related to the electric field strength ${\vec{E}}^{v}$, measured in a coordinate system referring to each point of a moving conductor, with velocity $\vec{v}$, with respect to the laboratory (1) [7], where $v^{2}\ll c^{2}$, and c is the speed of light, $\vec{B}$ is the magnetic induction, $\vec{a}$ is the magnetic vector-potential and φ is the electric scalar-potential, where

(1) $${\vec{E}}^{v}=\vec{E}+\vec{v}\times \vec{B}=\left(-\vec{grad}\;\varphi -\frac{\partial \vec{a}}{\partial t}\right)+\vec{v}\times \vec{B}.$$

**Figure 1:**
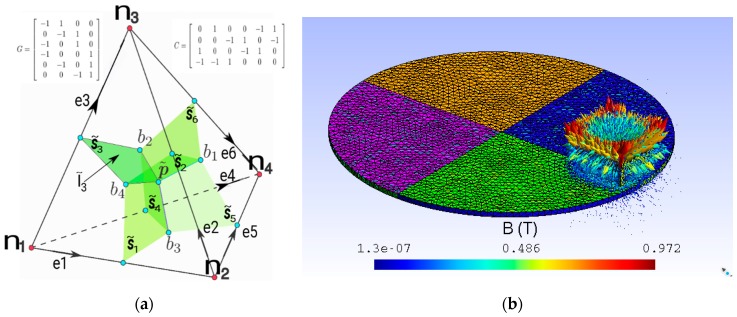
(**a**) Reference tetrahedron cell; (**b**) magnetic brake.

If we work with a permanent magnet, then $\frac{\partial \vec{a}}{\partial t}=0$, and (1) is simplified. The current density is shown in (2), where σ is the volumetric electric conductivity. The Equation (2) contains $\sigma \left(\vec{grad}\;\varphi \right)$. These are the electric currents produced by the corresponding electric potentials. This term of the equation is developed with CM in [8]. The first term of Equation (2) in CM is Equation (3). The electric currents refer to the electric conductivity constitutive matrix, $M_{\sigma }$. The differences of electric potentials are associated to the edges *e_i_*, *i* = 1:6, of the reference tetrahedron (see Figure 1a).

(2) $$\vec{j}=\sigma \left(-\vec{grad}\;\varphi +\vec{v}\times \vec{B}\right),$$

(3) $${\tilde{I}}_{\varphi }={\left[I_{i}\right]}_{6\times 1}^{\varphi }=M_{\sigma }{\left[U_{i}\right]}_{6\times 1}^{\varphi }=M_{\sigma }U_{\varphi },$$

where the matrix $M_{\sigma }=\frac{\sigma^{\epsilon }}{v^{\varepsilon }}{\tilde{S}}_{i}{\tilde{S}}_{j},\;i,\;j=1:6$, is function of the electric conductivity of each tetrahedron, its volume and the dot product of the surface vectors that correspond to the dual planes of primal edges *e_i_* and *e_j_* (see Figure 1a).

We start from the expression of the magnetic induction, (4), within each tetrahedron as a function of the magnetic fluxes associated with the faces of the reference tetrahedron [9].

The second term of (2), which corresponds to the Lorentz term, is developed in this article with CM, where

(4) $${\vec{B}}_{e}=\frac{1}{{3V}_{t}}\left({\vec{e}}_{4}\phi_{1}+{\vec{e}}_{5}\phi_{2}+{\vec{e}}_{6}\phi_{3}+\vec{0}\phi_{4}\right).$$

The integration of the differences of the electrical potential associated to both primal edges *e_i_*, *i* = 1:6, with the Lorentz term ${\vec{E}}_{Lo}={\vec{V}}_{e}\times {\vec{B}}_{e}$ is expressed in Equation (5):

(5) $$U_{i}=\int_{e_{i}}^{}\;{\vec{E}}_{Lo}\cdot d\vec{l}=\int_{e_{i}}^{}\left({\vec{V}}_{e}\times {\vec{B}}_{e}\right)\cdot d\vec{l}$$

Solving (5), the $U_{i}$ in the primal edges *e_i_* is expressed in (6),

(6) $$U_{i}=\frac{1}{{3V}_{t}}\left({\vec{V}}_{e}\times {\vec{e}}_{4}\cdot {\vec{e}}_{i}\phi_{1}+{\vec{V}}_{e}\times {\vec{e}}_{5}\cdot {\vec{e}}_{i}\phi_{2}+{\vec{V}}_{e}\times {\vec{e}}_{6}\cdot {\vec{e}}_{i}\phi_{3}+\vec{0}\phi_{4}\right),$$

where

(7) $${\vec{V}}_{e}\times {\vec{e}}_{e}=\left|\begin{array}{ccc} \vec{i} & \vec{j} & \vec{k}\\ V_{x}^{e} & V_{y}^{e} & V_{z}^{e}\\ e_{x} & e_{y} & e_{z} \end{array}\right|,$$

(8) $${\vec{e}}_{i}\cdot {\vec{e}}_{j}=e_{x}^{i}e_{x}^{j}+e_{y}^{i}e_{y}^{j}+e_{z}^{i}e_{z}^{j}.$$

With the six edges of the reference tetrahedron, the Lorentz voltages-vector is shown in (9) and $V_{Lo}$ is calculated using (10).

(9) $$U_{Lo}={\left[U_{i}\right]}_{6\times 1}^{L_{o}}=V_{Lo}{\left[\phi_{i}\right]}_{4\times 1}^{}.$$

(10) $$V_{Lo}=\frac{1}{{3V}_{t}}\left[\begin{array}{cccc} \begin{array}{c} {\vec{V}}_{e}\times {\vec{e}}_{4}\cdot {\vec{e}}_{1}\\ {\vec{V}}_{e}\times {\vec{e}}_{4}\cdot {\vec{e}}_{2}\\ {\vec{V}}_{e}\times {\vec{e}}_{4}\cdot {\vec{e}}_{3} \end{array} & \begin{array}{c} {\vec{V}}_{e}\times {\vec{e}}_{5}\cdot {\vec{e}}_{1}\\ {\vec{V}}_{e}\times {\vec{e}}_{5}\cdot {\vec{e}}_{2}\\ {\vec{V}}_{e}\times {\vec{e}}_{5}\cdot {\vec{e}}_{3} \end{array} & \begin{array}{c} {\vec{V}}_{e}\times {\vec{e}}_{6}\cdot {\vec{e}}_{1}\\ {\vec{V}}_{e}\times {\vec{e}}_{6}\cdot {\vec{e}}_{2}\\ {\vec{V}}_{e}\times {\vec{e}}_{6}\cdot {\vec{e}}_{3} \end{array} & \begin{array}{c} 0\\ 0\\ 0 \end{array}\\ \begin{array}{c} {\vec{V}}_{e}\times {\vec{e}}_{4}\cdot {\vec{e}}_{4}\\ {\vec{V}}_{e}\times {\vec{e}}_{4}\cdot {\vec{e}}_{5}\\ {\vec{V}}_{e}\times {\vec{e}}_{4}\cdot {\vec{e}}_{6} \end{array} & \begin{array}{c} {\vec{V}}_{e}\times {\vec{e}}_{5}\cdot {\vec{e}}_{4}\\ {\vec{V}}_{e}\times {\vec{e}}_{5}\cdot {\vec{e}}_{5}\\ {\vec{V}}_{e}\times {\vec{e}}_{5}\cdot {\vec{e}}_{6} \end{array} & \begin{array}{c} {\vec{V}}_{e}\times {\vec{e}}_{6}\cdot {\vec{e}}_{4}\\ {\vec{V}}_{e}\times {\vec{e}}_{6}\cdot {\vec{e}}_{5}\\ {\vec{V}}_{e}\times {\vec{e}}_{6}\cdot {\vec{e}}_{6} \end{array} & \begin{array}{c} 0\\ 0\\ 0 \end{array} \end{array}\right].$$

In the reference tetrahedron, the magnetic fluxes can be expressed as magnetic potentials associated to the edges of the tetrahedron [6]. This is developed as (11),

(11) $${\left[\phi_{i}\right]}_{4\times 1}^{}={\left[C\right]}_{4\times 6}^{}{\left[a_{i}\right]}_{6\times 1}^{}.$$

Substituting (11) in (9), we get (12), which is the proposed equation in this work. Matrix C is the incidence matrix between the faces and edges of the reference tetrahedron (see Figure 1a),

(12) $$U_{Lo}={\left[U_{i}\right]}_{6\times 1}^{L_{o}}=V_{Lo}C{\left[a_{i}\right]}_{6\times 1}^{}=V_{Lo}Ca.$$

The current produced by the Lorentz voltage is shown in (13). The total current will be $\tilde{I}={\tilde{I}}_{\phi }+{\tilde{I}}_{Lo}$:

(13) $${\tilde{I}}_{Lo}=M_{\sigma }U_{Lo}=M_{\sigma }V_{Lo}Ca=MV_{L}a,$$

where *MV_L_* is the new constitutive matrix proposed for the calculation of the Lorentz current, *I_Lo_*, depending on magnetic potentials.

To validate the proposed equation, the problem of the magnetic brake (MB) is posed using FF. The MB is a disk, made of copper, which rotates around an orthogonal axis, *y*. On the upper side of the disk there is a neodymium permanent magnet (see Figure 1b). The velocity for any point of the disk is calculated by (14), where $\vec{W_{r}}$ is the angular velocity of the disk and $\vec{r}$ is the radius-vector respective to the central point of the disk,

(14) $${\vec{V}}_{e}=\vec{W_{r}}\times \vec{r}.$$

To solve the problem, the electric-potential φ and a magnetic-potential formulation are used. In this formulation, the continuity equation for electric current, (15), is used, where $\tilde{D}$ is the face-volume incidence matrix in the dual mesh,

(15) $$\tilde{D}\tilde{I}=\tilde{D}\left({\tilde{I}}_{\varphi }+{\tilde{I}}_{Lo}\right)=0.$$

Taking into account the fact that $\tilde{D}=-G^{t}$, where G is the edge-node incidence matrix in the primal mesh (see Figure 1a), then Equations (3), (12) and (13), with the continuity equation for electric current, and all terms—including the term $\left(\frac{\partial \vec{a}}{\partial t}\right)$—remain as indicated in (16):

(16) $${-G}^{t}M_{\sigma }jWa+G^{t}{MV}_{L}a-G^{t}M_{\sigma }G\varphi =0.$$

The definition equation for the permanent magnet is Equation (17). $M_{\nu }$ is the magnetic reluctivity matrix [4], ${\tilde{F}}_{c}$ is the vector of coercive magnetomotive forces supplied by the manufacturer of the magnet, and $\tilde{F}$ is the vector of magnetomotive forces associated to the edges of the dual mesh:

(17) $$\tilde{F}=M_{\nu }\phi -{\tilde{F}}_{e}.$$

The constitutive equation in the air and the conductive material is (18). If Ampere’s law (19) is applied to the permanent magnet, then it reads as (20). If the same law is applied to the air, it is shown as (21). Finally, applying Ampere’s law to the conductive material, then we obtain (22),

(18) $$\tilde{F}=M_{\nu }\phi ,$$

(19) $$\tilde{C}\tilde{F}=\tilde{I},$$

(20) $$\tilde{C}\left(M_{\nu }\phi -{\tilde{F}}_{e}\right)=0,$$

(21) $${\tilde{C}M}_{\nu }\phi =0,$$

(22) $${\tilde{C}M}_{\nu }\phi =M_{\sigma }\left(-G\varphi -\frac{\partial a}{\partial t}+V_{Lo}\phi \right),$$

where $\tilde{C}$ is the discrete curl operator in the dual mesh. Taking into account the fact that $\tilde{C}=C^{t}$ and ϕ=Ca, then Ampere’s law and continuity equation for electric current in all domains both form a system of equations as in (23). This system of equations, in a sinusoidal steady state, has been programmed in C++, using the numerical software package PETSc [10].

(23) $$\left[\begin{array}{cc} C^{t}M_{\nu }C-{MV}_{L}+M_{\sigma }jW & M_{\sigma }G\\ {-G}^{t}M_{\sigma }jW+G^{t}{MV}_{L} & {-G}^{t}M_{\sigma }G \end{array}\right]\left[\begin{array}{c} a\\ \varphi \end{array}\right]=\left[\begin{array}{c} C^{t}{\tilde{F}}_{e}\\ 0 \end{array}\right].$$

## 3. Results and Discussion

### 3.1. Characterization of the Magnetic Brake

**Figure 2:**
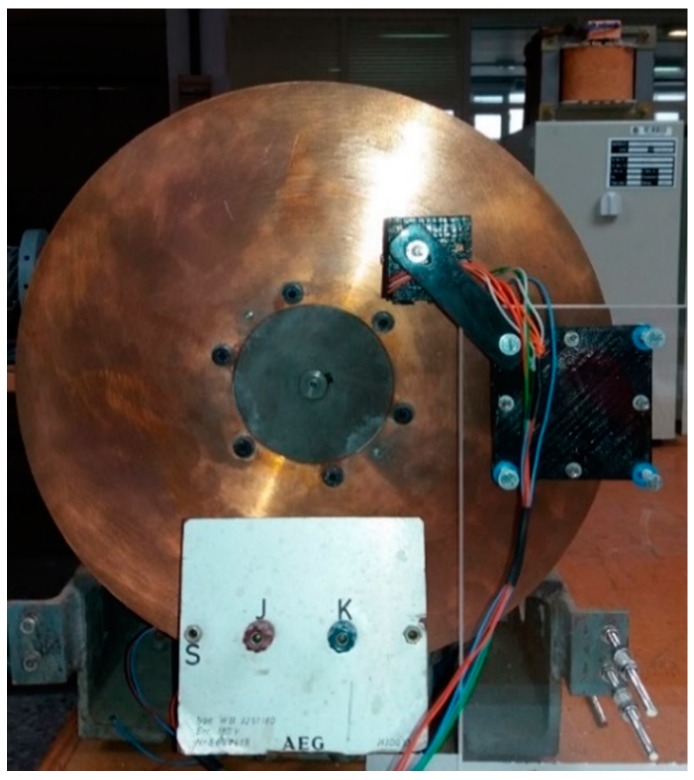
Disk, magnet and Hall sensors, frontal view.

**Figure 3:**
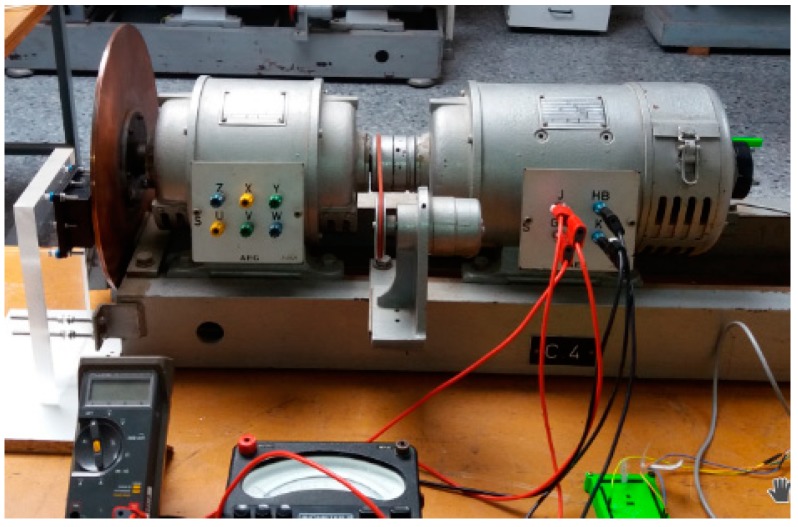
Magnetic brake and DC motor.

The magnetic brake consists of a disk of copper alloy and a permanent magnet located at a distance *d* (see Figure 2). The disk is coupled to a DC motor. The DC motor rotates at angular velocity ${\vec{W}}_{r}$ (see Figure 3).

The permanent magnet, which is used in the numerical simulations and experiments at the laboratory, is modeled in the second quadrant, with H<0 and B>0. It is a linear model with two parameters, $B_{r}$ and μ. This model is adequate when rare earth permanents magnets are used. The characteristics of the permanent magnet are the following: the material us NdFeB, with a block form of 50.8 × 50.8 × 25.4 mm, a magnetization sense in axis *y* along the dimension 25.4 mm, the coating is nickel-plated (Ni-Cu-Ni), manufactured by sintering, the magnetization type is N40, the remanence $B_{r}$ is in the interval 1.26–1.29 T, the coercive $H_{c}$ is in the interval 860–955 kA/m, the intrinsic coercive $H_{c}\geq$ 955 kA/m and the maximum energetic product is in the interval 303–318 kJ/m^3^.

The disk has a volumetric electric conductivity of σ= 4.1·10^7^ S/m, its diameter is 315 mm and its thickness is 5 mm.

When the equation system in (23) has been solved, the losses by Joule effect are obtained in the disk. These are calculated using a volume-integral as in (24),

(24) $$P_{J}=\int \frac{1}{\sigma }\vec{J}\cdot \vec{J}\;dv,$$

where $\vec{J}$ is the volumetric density electric current indicated in (22). The braking torque, along the *y* axis, is calculated using a volume-integral as indicated in (25).

(25) $$\vec{T}=\int \left(\vec{r}\times \vec{J}\times \vec{B}\right)dv.$$

### 3.2. Numerical Simulations

Different numerical experiments have been developed using different angular velocities with an air-gap between the disk and the permanent magnet equivalent to 6 mm. The constitutive matrix of velocities proposed in (13), developed in CM and Equations (23), have been used in these simulations. The arising system, Equation (23), is not as symmetric as in FEM. We use numerical methods based in the subspace of Kryslov due to the fact that the matrices are sparse and of large dimensions. These algorithms are implemented in the software PETSc [10].

In particular, the linear solver employed is the generalized minimal residual algorithm (GMRES). Besides this, we precondition the matrix to improve the condition number of the matrix to reduce the number of iterations. This method is valid for non-symmetric systems. Relative and absolute tolerances with an order of magnitude of 10^−10^ are enough to achieve convergence for the linear solver.

We have used tetrahedral elements because they are better for complex geometries. The number of elements depends on the model analyzed. For example, in a particular implementation, we used 261,868 elements, and the assembling and solution time was lower than 500 s, with an Intel core machine i7-3820, 3.6GHz and 32GB of RAM with four cores and eight threads of execution. The convergence has been studied, increasing the number of elements until a reasonable rate of convergence was obtained.

To avoid singularities in the problem solution, we control the dimensionless Péclet number to get a reasonable convergence, with manual mesh refinements, using a higher density of points where the linear velocity of the disk is higher.

**Figure 4:**
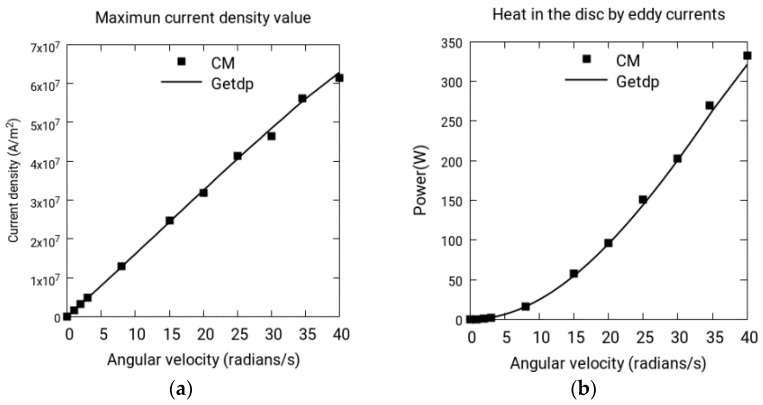
(**a**) Maximum current density in the disk, CM vs. Getdp; (**b**) the heat dissipated in the disk, CM vs. Getdp.

The results obtained for the disk of copper alloy are shown in Figure 4a,b. In Figure 4a, the maximum current density in the disk is compared in CM and software package Getdp [11]. In Figure 4b, the losses by Joule’s effect are compared. The proposed numerical simulations have been developed in CM. The reference, used to compare the results, is Getdp.

**Figure 5:**
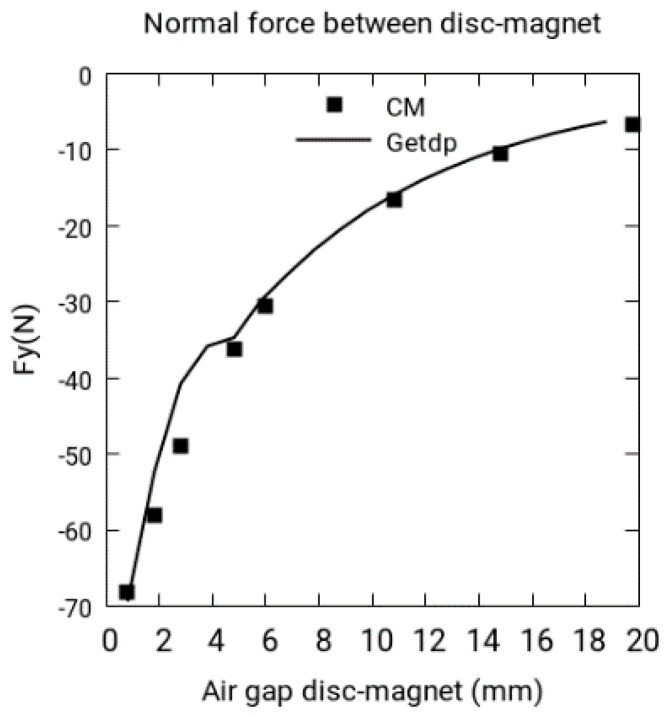
Normal force *Fy* with *Wr* = 34.55 rad/s, cell method (CM) vs. Getdp.

The software package Gmsh [12] is used in the mesh and the data visualization. In Figure 5, the normal force between the disk and permanent magnet is shown. The results obtained in CM are compared with the results obtained in FEM.

### 3.3. Numerical Validation of the Simulations

Three experiments were developed and the results are compared with those obtained through CM and Getdp. These experiments are specified in Table 1.

The statistics used in the validation of the models are the following: R^2^, determination coefficient, see [13,14,15]; MSE: mean square error, see [13,16,17,18,19]; RMSE: root mean square error, see [18,19,20]; RMSPE: root M. S. perceptual error, see [21]; MAE: mean absolute error, see [16,18,20,22]; MAEP: mean absolute percentage error, see [21]; PBIAS, percentage bias, see [13,16,23,24]. NSEF: modelling efficiency Nash & Sutcliffe, see [16,17,19]; U1: Theil inequality coefficient, see [24,25,26]; U^M^: bias proportion or difference between means (systematic error), see [25]; U^S^: variance proportion (systematic error), see [25]; U^C^: covariance proportion (non-systematic error), see [25]. d: d-Willmott Index, see [27]; MEF: modelling efficiency, see [28]; CD: determination coefficient of modelling, see [28]; C: error coefficient of modelling, see [28].

Table 2 shows the metrics of the comparisons that have been proposed in Table 1, following the statistics previously mentioned.

In Table 2, we can see that all the metrics are different from the optimum. MAE is given in true measurements with its units. This metrics show the validity of FF-CM for our analysis.

To understand the differences of magnitude in MSE, RMSE and MAE, we have to observe that C1 is a large quantity—with a current density on the order of magnitude 10^7^ (see Figure 4a)—and both C2 and C3 are small—with a heat in the disk and normal force on the order of magnitude 10^2^ (see Figure 4b and Figure 5). Therefore, C1 is apparently a large quantity and both C2 and C3 are small quantities. However, as these quantities are absolute errors they seem large. The optimum is 0 because this is the limit to which these magnitudes tend as we increase the number of elements in the mesh.

Table 3 presents the evolution of the error and the execution time for the assembling and solution for Joule heating as the number of degrees of freedom increases in the system of Equations (23) for an angular velocity of 15 rad/s. With 46,723 degrees of freedom, we obtain an error less than 3%, but we have implemented, in this particular case, 233,471 degrees of freedom obtaining a negligible error in 324.30 s.

### 3.4. Experimental Validation of the Simulation

In this section, we obtain experimental measures to validate the cell method used in the simulation of the magnetic field in the proximities of a magnetic brake. Besides this, we experimentally analyze the electromagnetic torque generated in the brake.

In order to develop the experimental system, it is necessary to calibrate the Hall sensors that measure the magnetic field. These sensors will be previously calibrated through reference magnets.

#### 3.4.1. Calibration of the Hall Sensors

First of all, we will characterize the reference magnets, and then we will calculate the gain of the Hall sensors. For the measurements, we have used precision weights that have been verified in a precision balance showing a negligible deviation. Distances have been measured with a precision of hundredth of a millimeter, which is a very good precision for our experiments.

Reference magnet characterization: The experimental measurement of the magnetic field on the proximities of the copper disk is performed with three Hall sensors that allow the measurement of the magnetic induction in the three directions of the Cartesian axes *x*, *y*, *z*. These sensors are of the 49E linear type in a measurement range between −90 mT and +90 mT [29].

**Figure 6:**
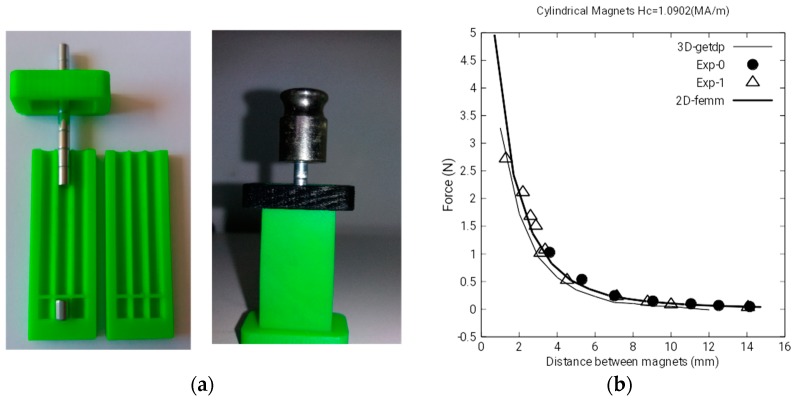
(**a**) Magnet arrangement with magnetic and mechanical equilibrium force; (**b**) reference magnet characterization.

In order to calculate the gain of each sensor, we use a consistent magnetic field source with two magnets of NdFeB of cylindrical shape and a radius of 2 mm and a height of 5 and 40 mm, respectively, as shown in Figure 6a.

The first set of measurements is performed in order to determine, in an accurate way, the intensity of the coercitivity field, *H_c_*, of the magnets, because it is unknown.

In this way, the two magnets are located as in Figure 6a, and the equilibrium force between both is measured for different distances between 2 and 14 mm. The mass used to obtain the equilibrium force consists of different precision weights from 2 to 100 g and are situated on the 40 mm magnet as can be observed in Figure 6a. We have taken two sets of experimental data to assure the repeatability of the measures and they are called Exp-0 and Exp-1, as shown in Figure 6b.

The intensity of the coercitivity field, *H_c_*, which better fits the data, is *H_c_* = 1.0902 MA/m. These data have been obtained by successive MEF simulations in 2D and 3D, finding the repulsion force between the magnets. Figure 6b shows the best fits obtained through MEF using Getdp 3D software and femm 2D software which takes advantage of the axial symmetries of this problem. Besides this, working in 2D, we can use denser meshes for the same computational cost, obtaining more accurate results.

These fits confirm that MEF is an adequate tool to calculate the magnetic field in the proximities of magnetic fields generated by permanent magnets.

Calculation of the gain of the Hall sensors: Although we use three Hall sensors of the same type and manufacturer [30], the gain of each one is slightly different. In this section, we experimentally characterize the gain of each one.

**Figure 7:**
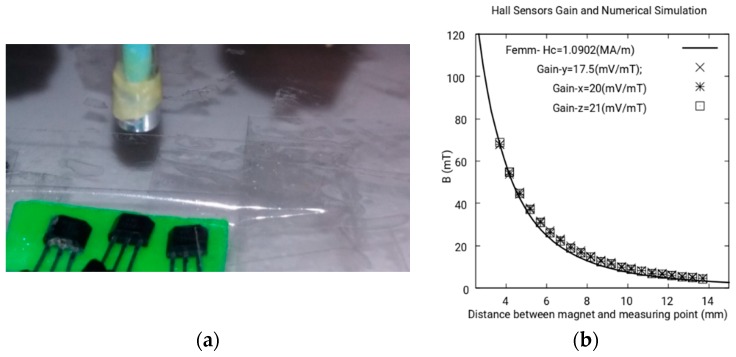
(**a**) CNC tip on the Hall sensors; (**b**) calculation of the gain of the Hall sensors.

As Figure 7a shows, we locate the magnet using a high precision CNC―Computer Numerical Control―at a particular distance from the Hall sensor. Measuring the electric potential difference in the Hall sensor generated by the magnetic field of the magnet, we can determinate the gain of the sensor. The magnetic field was calculated using femm MEF software as explained in the previous section.

The gains obtained for Hall sensors *x*, *y*, *z* are 20.0, 17.5, and 21 mV/mT, respectively, as can be seen in Figure 7b.

#### 3.4.2. Measures of the Magnetic Field and Torque

As previously mentioned, we use a disk made of a copper alloy, of diameter 315 mm and thickness 5 mm that can rotate in both directions. This disk is moved by a DC motor regulated in speed by the electric potential of the armature.

**Figure 8:**
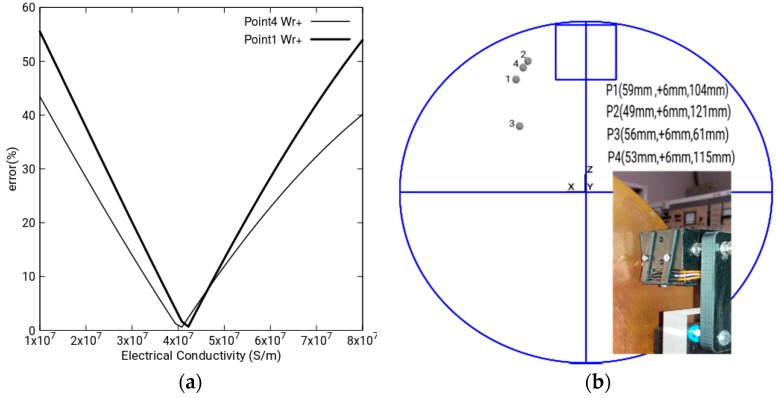
(**a**) Optimization of copper alloy conductivity; (**b**) position of the Hall sensors on the disk and position of the four points where the magnetic field vector has been measured.

The effective conductivity of the copper alloy has been obtained by minimizing the error between the measured and simulated magnetic field, varying the electric conductivity between 1 × 10^7^ and 8 × 10^7^ S/m, with an angular velocity of the disk of 20.49 rad/s, as Figure 8a shows. The obtained value for a minimum error corresponds to 4.1 × 10^7^ S/m. With this value, we have implemented all the numeric simulations from now on.

At a variable distance from the disk and with magnetization in the *y* direction, we have placed a permanent magnet. The magnet is a brick with the dimensions 50.8 × 50.8 × 25.4 mm. As shown in Figure 8b, we have also placed three Hall sensors in a trihedral trirectangle which determines a unique vertex. The trihedral can be displaced in the *x*, *y*, *z* directions.

Then, we analyze the magnetic field vector as a function of the angular velocity of the disk in the points 1, 2, 3 and 4, as can be seen in Figure 8b.

Experimental results and magnetic field discussion: We have taken eight measurements for different values of the angular velocity of the magnetic field in points 1, 2, 3 and 4 of Figure 8b. Four have been taken with the disk rotating clockwise and four anticlockwise.

**Figure 9:**
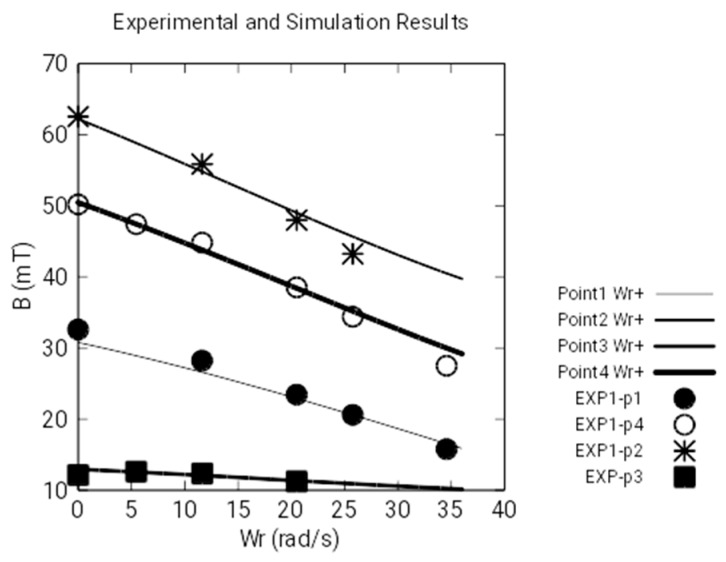
Measurement of the magnetic field with clockwise *Wr*–.

Figure 9 represents the module of magnetic induction measured by the Hall transducers and the numeric simulations of the angular anticlockwise velocity, *Wr*–. We observe that there is a great coincidence between experimental values and those obtained by numeric simulation because the discrete values are almost coincident with the continuous curves that represent the simulations. Besides this, the curves are increasing: that is to say, the magnetic induction increases as the angular velocity increases.

**Figure 10:**
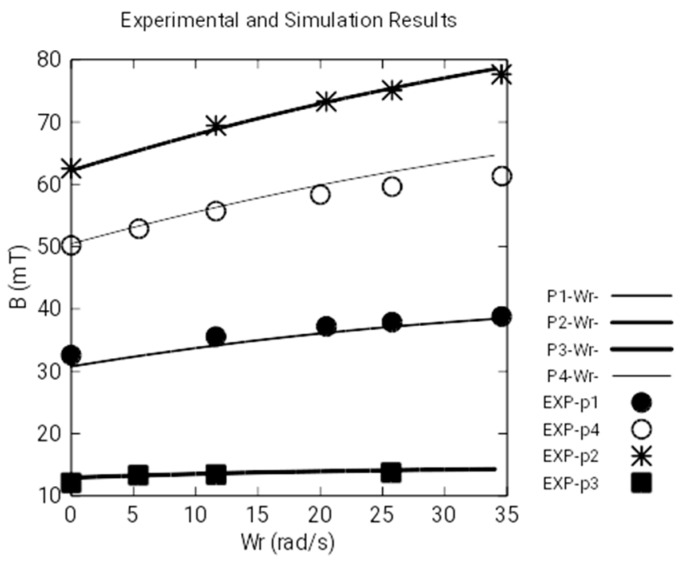
Measurement of the magnetic field with clockwise *Wr+*.

**Figure 11:**
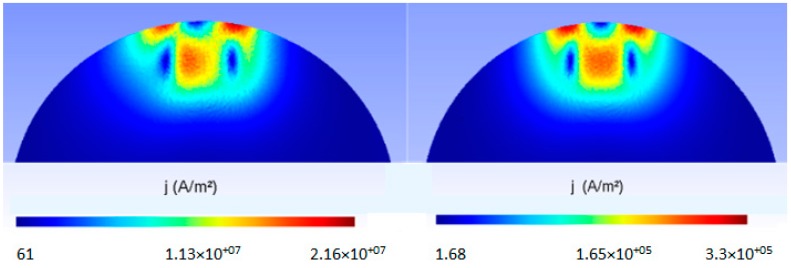
Module of the induced current densities for *Wr*– = 34 and 0.5 rad/s, respectively.

On the other hand, Figure 10 represents the module of the magnetic induction, measured with the Hall sensors at the same points, and the numeric simulations, as functions of the angular clockwise velocity, *Wr+*. Likewise, we observe that there is a great coincidence between the experimental values and those obtained by numeric simulation. However, these curves are decreasing: that is to say, the magnetic induction decreases as the angular velocity increases. This behavior is due to the asymmetry of the currents induced in the disk when the rotating direction of the angular velocity changes (see Figure 11).

**Figure 12:**
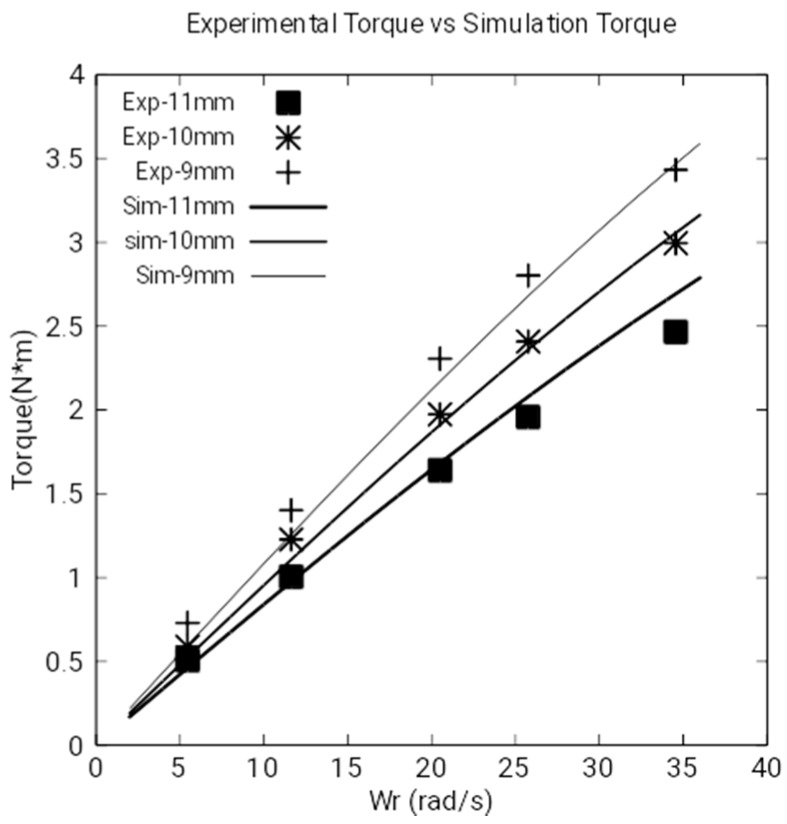
Experimental and simulated results of the electromagnetic torque in the disk.

Experimental results and electromagnetic torque: This set of experiments consists of the measurements of the losses by the Joule effect made in the laboratory. These measurements have been obtained by subtracting the total electrical power at the input of the DC motor from the Joule losses in the armature windings and the mechanical losses in the non-load regimen, without the magnetic brake. With these calculations, the electromagnetic torque for braking is obtained for a determinate angular velocity (see Figure 12). The characteristics of the DC motor are the following: AEG, direct current, armature 220 V–2.2 A, field 220 V, 0.5 kW, 1400 rpm, IP E22.

Figure 12 shows the electromagnetic torque obtained by experimentation and simulation in the copper disk for an angular velocity from 0 to 35 rad/s.

The magnets are situated at distances of 9, 10 and 11 mm. Experimental results are almost coincident with numeric simulations. Besides this, as expected, we can observe that as the angular velocity increases, the torque increases. It can also be observed that as the proximity to the disk increases, the torque also increases.

As there is no induced current when the angular velocity is zero, the magnetic torque converges to zero in both experimental and simulation results.

Figure 9, Figure 10 and Figure 12 show the experimental and simulation results. As can be seen, the differences are small. Figure 9 and Figure 10 show the calculations and measurements of magnetic fields and Figure 12 shows the calculations and measurements of the electromagnetic torque.

## 4. Conclusions

A constitutive matrix has been developed into the cell method in 3D. The Lorenz electric current has been obtained using the magnetic potential.

This methodology has been applied to a disk that moves inside a magnetic field.

The results obtained have been compared with the finite element method, using the free software packages Getdp and femm as a reference. The error is less than 0.1173%.

A second verification has been made in the laboratory using Hall sensors to measure the magnetic field in the proximity of the magnetic brake.

## Figures and Tables

**Table 1:** Numerical experiments developed.

**C1.**	Maximum current density value (see Figure 4a)
**C2:**	Heat in the disk by eddy currents (see Figure 4b)
**C3:**	Normal force between disk-magnet (see Figure 5)

**Table 2:** Metrics of the comparisons proposed.

Comparatives	C1	C2	C3
R^2^ [0, +1] Optimum: +1	0.9990	0.9998	0.9760
MSE [0, +∞] Optimum: 0	2.25 ×10^11^	17.4812	17.4794
RMSE [uds]	0.47 × 10^6^	4.1810	4.1808
RMSPE [−1, +1] Optimum: 0	0.0386	0.0500	−0.1173
MAE [uds]	6 × 10^5^	2.3796	3.1703
MAEP [−1, +1] Optimum: 0	0.0257	0.0284	−0.0889
PBIAS [−1, +1] Optimum: 0	−0.0116	0.0276	0.0741
NSEF [−∞, 1] Optimum: 1	0.9982	0.9984	0.9526
U1 de Theil [0, 1] Optimum: 0	0.0144	0.0155	0.0499
UM de Theil [0, 1] Optimum: 0	0.0890	0.3239	0.4658
US de Theil [0, 1] Optimum: 0	0.3489	0.5454	0.0115
UC de Theil [0, 1] Optimum: 0	0.5621	0.1307	0.5227
Willmott-dW [0, 1] Optimum: 1	0.9995	0.9996	0.9884
MEF [−∞, 1] Optimum: 1	0.9982	0.9984	0.9526
CD [−∞, +∞] Optimum: 1	1.0516	0.9424	0.9352
C [−∞, +∞] Optimum: 0	0.0257	0.0284	−0.0889

**Table 3:** Evolution of the error and the execution time.

Degrees of Freedom	Time (s)	Joule Heating (W)	Error (%)
3018	0.42	76.62	42.90
4718	0.85	71.92	34.10
22,796	7.86	55.82	4.10
34,466	15.00	55.58	3.69
46,723	24.65	55.20	2.98
61,937	37.90	54.90	2.42
86,127	74.44	54.84	2.31
132,518	138.18	53.90	0.55
233,471	324.30	53.60	0.00

## Nomenclature

**Symbol**	**Name**	**Unit**
$\vec{E}$	Electric field strength	V/m
${\vec{E}}^{v}$	Electric field strength with velocity *v*	V/m
*ϕ*	Electric scalar-potential	V
*σ*	Electric conductivity	S/m
${\tilde{I}}_{\varphi }$	Electric currents by electric scalar-potential	A
*M_σ_*	Electric conductivity constitutive matrix	S
$U_{\varphi }$	Electric potential differences	V
*σ^t^*	Electric conductivity of the tetrahedron	S/m
$\vec{B}$	Magnetic induction	Wb/m^2^
*φ*	Magnetic flux	Wb
*a*	Magnetic potential in primal edges	Wb
$\vec{a}$	Magnetic vector-potential	Wb/m
*C*, $\tilde{C}$	Incidence matrix faces-edges in primal and dual mesh	-
$\tilde{D}$	Incidence matrix face-volume of dual mesh	-
*G*	Incidence matrix edges-nodes of primal mesh	-
${\vec{E}}_{Lo}$	Lorentz electric field strength	V/m
*U_Lo_*	Vector of Lorentz tension in primal edges	V
*V_Lo_*	Vector of Lorentz	s^−1^
${\tilde{I}}_{Lo}$	Vector of Lorentz currents in dual face	A
*MV_L_*	New constitutive matrix of Lorentz	S/s
*M_ν_*, *M_σ_*	Magnetic and electric constitutive matrix	A/Wb
*W*	Angular frequency	1/s
$\vec{v}$	Lineal velocity	m/s
$\vec{W_{r}}$	Angular velocity	1/s
*t*	Time	s
$\vec{J}$	Current density	A/m^2^
$\vec{e_{i}}$	Length edge of *i* vector	m
*V_t_*	Volume of tetrahedron	m^3^
${\vec{V}}_{e}$	Barycenter velocity of the tetrahedron	m/s
$\vec{i},\vec{j},\vec{k}$	Unitary vectors	-
${\tilde{S}}_{i}$	*i* surface vector of dual mesh	m^2^
$\vec{r}$	Radial vector pointing from the center of the disk	m
${\tilde{F}}_{e}$	Coercive magnetomotive forces of the magnet	A
$\tilde{F}$	Vector of magnetomotive forces of the dual mesh edges	A
*i*	Imaginary unit	-
*P_j_*	Joule heating	W
*T_j_*	Braking torque	Nm
